# Comprehensive analysis of the rostral and caudal cerebral artery branching patterns in the dromedary camel (*Camelus dromedarius*)

**DOI:** 10.3389/fvets.2024.1426372

**Published:** 2024-07-19

**Authors:** Ahmad Al Aiyan, Rinsha Balan

**Affiliations:** College of Agriculture and Veterinary Medicine, Department of Veterinary Medicine, United Arab Emirates University, Abu Dhabi, United Arab Emirates

**Keywords:** dromedary camel, rostral cerebral artery, caudal cerebral artery, RERM, casting techniques

## Abstract

**Introduction:**

In mammals, the cerebral cortex depends on a robust blood supply for optimal function. The rostral and caudal cerebral arteries are critical for supplying the cerebrum. This study presents the first detailed anatomical description of the rostral and caudal cerebral arteries of dromedary camels (*Camelus dromedarius*), including their origins, routes, and complex branching patterns.

**Methods:**

A sample of 55 heads from male dromedary camels aged 2–6 years was analyzed using advanced casting techniques with various casting materials to provide precise visualization of these arterial structures.

**Results:**

The rostral cerebral arteries originate dorsally from the rostral epidural rete mirabile (RERM), while the caudal cerebral arteries arise from the caudal communicating artery, which is another branch of the RERM. Both sets of arteries give rise to multiple cortical branches responsible for supplying the medial aspects of the frontal, parietal, and temporal lobes, as well as the medial and caudal regions of the occipital lobes.

**Conclusion:**

This study significantly expands our understanding of the cerebrovascular anatomy of dromedary camels. Our findings have potential implications for veterinary medicine in the diagnosis and treatment of neurological disorders in camels and may offer insights into broader comparative neuroscience research.

## 1 Introduction

The cerebral tissue is supplied by multiple blood vessels originating from different sources of incoming blood flow through the carotid and vertebrobasilar systems. Collateral circulation serves as a protective shield against ischemia and stroke, as a blockage in one set of afferent arteries is countered by compensating for blood flow from a different collateral artery (1, 2). As shown in the cerebral arterial circle called Circle of Willis, a viable adaptive strategy for preventing ischemia is to pool blood from various afferent systems before supplying it to the brain. Blood from the carotid and vertebral systems mixes through an anastomosing blood vessel known as the cerebral arterial circle at the base of the cranial cavity. The arterial circle supplies various brain structures through the blood vessels (3, 4). Further, most artiodactyls, including cattle, sheep, goats, pigs, and carnivores, possess an extensive network of anastomosing arteries called the rostral epidural rete mirabile (RERM), formed by the internal carotid arteries and rostral branches of the maxillary arteries (5–8). In addition to reducing cerebral blood pressure, the RERM dissipates heat from warm arterial blood by lying in a pool of cooler venous blood, lowering the temperature of the blood that reaches the brain (9–11).

A pair of major arteries, the caudal communicating (CCoA) and rostral cerebral arteries (RCA) emerge from the RERM, forming the cerebral arterial circle after joining the basilar artery caudally. The cerebral arterial circle gives rise to multiple branches that supply blood to different areas of the dromedary brain, including the cerebrum, cerebellum, and brainstem (12).

Three primary cerebral arteries supply blood to the cerebrum: the rostral cerebral (RCA), middle cerebral (MCA), and caudal cerebral (CCA) arteries. These three main cerebral arteries ensure a constant and adequate blood supply to meet the metabolic demands of the cerebrum, which is considered the largest part of the brain and is responsible for various cognitive functions (13). Despite previous studies on the general cerebral circulation in camels, a detailed understanding of the specific branching patterns of the rostral and caudal cerebral arteries remains undefined. Although some studies have mentioned these arteries as part of the overall blood supply to the brain of animals, they have not discussed their branching pattern, course, or anastomosis in detail, limiting our understanding of their specific roles and potential clinical implications. A comprehensive map of the branches of the rostral and caudal cerebral arteries will provide crucial anatomical information for veterinary clinicians, facilitating precise diagnosis and targeted treatment of neurological disorders. Despite the recent technological advancements in veterinary anatomy, the importance of gross anatomy for research remains fundamental (14).

The primary objective of this study was to bridge the existing knowledge gap regarding the branching patterns of the RCA and CCA. By employing advanced casting procedures, we were able to generate accurate three-dimensional representations of the cerebral arteries, thereby highlighting minor branches that might otherwise be overlooked during dissection. This study sought to offer a thorough representation of the RCA and CCA, their branches, anatomical paths within the brain, and the areas of the brain they supply in the one-humped camel. The findings of this study will be an essential asset for anatomical and physiological researchers and contribute significantly to our understanding of cerebral arterial architecture in dromedaries.

## 2 Materials and methods

This study was conducted in accordance with the Research Ethics Policy established by the United Arab Emirates University and was approved by the Animal Research Ethics Committee (ERA_2019_5850). Heads of 55 male Omani dromedaries were sourced from the Al Khazna, Abu Dhabi Food Control Authority (ADFCA), and Bawadi, Al Ain city municipal Camel Slaughterhouses. The studied dromedaries ranged in age from 2–6 years. Each head included a neck segment containing three to seven vertebrae. The common carotid arteries were cannulated to inject the casting agent.

Thirty heads were cast using Epoxy Resin (Gulfguard Epoxy), twenty heads were cast using Polyurethane Resin (Polytek EasyFlo 60 Liquid Plastic), and five heads were cast using Latex Neoprene (Latex Globalsil AL 20). The volume administered varied between 400 and 800 mL, as determined by the head size and the number of cervical vertebrae (15, 16). The casting materials were injected manually using 60 mL syringes. Following injection, the heads were refrigerated at 5°C for at least 24 h to solidify the casting agent. Using a rotating power saw (DeWalt DWE4001 with a DeWalt 100 × 0 × 16 mm blade), the cranial roof and vertebrae were opened. Our study utilized epoxy resin for its ability to create a highly rigid cast, which was ideal for obtaining a detailed 3D representation of blood vessels in the brain. In 20 samples, high-pressure water was used to remove the brain tissue, leaving the epoxy/polyurethane casts intact. In 10 cases, potassium hydroxide solution (5%) was used to remove non-cast tissues, including bone. Sodium carbonate solution at 60°C was used to digest only the soft tissues while preserving the bony structures and cerebral arteries. Finally, five heads injected with latex were fixed in formaldehyde (6%), followed by careful dissection and extraction of the brain and arterial supply.

## 3 Results

**Figure 1:**
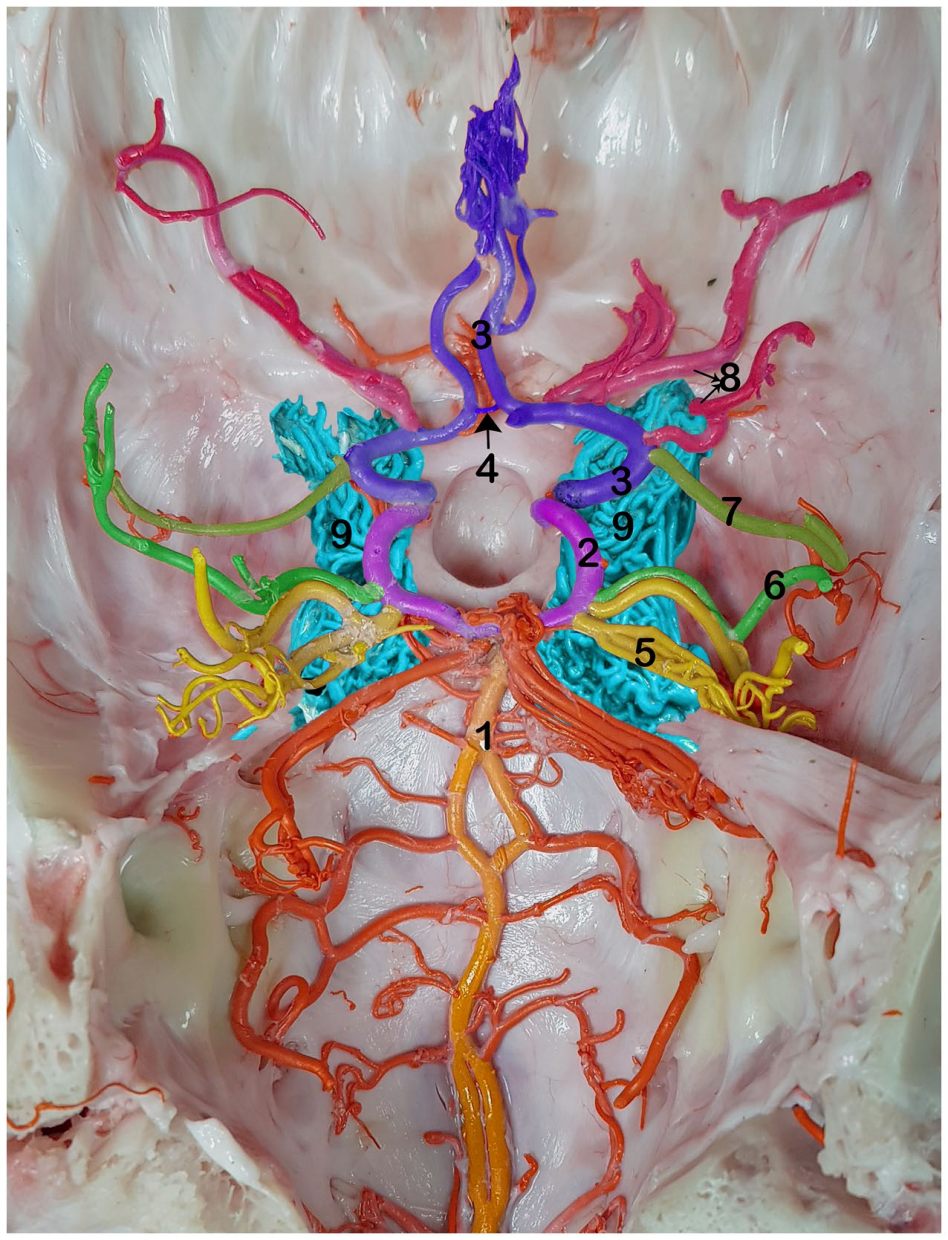
Dorsal view of the RERM with cerebral arterial circle beneath it in the dromedary brain 1, basilar artery; 2, caudal communicating artery; 3, rostral cerebral artery; 4, rostral communicating artery; 5, caudal cerebral artery; 6, caudal choroidal artery; 7, rostral choroidal artery; 8, middle cerebral artery; 9, RERM.

The cerebral arterial circle (CAC) is a circular structure that collects incoming blood from the rostral epidural rete mirabile (RERM) and basilar artery and then distributes it to the brain. A single basilar artery, two caudal communicating arteries (CCoA), and two rostral cerebral arteries (RCA) were anastomosed in the form of an hourglass-shaped arterial circle. The lateral wall of the CAC is formed by the paired RCA in the rostral half and the paired CCoA in the caudal half, providing the main source of blood for the CAC (Figure 1).

**Figure 2:**
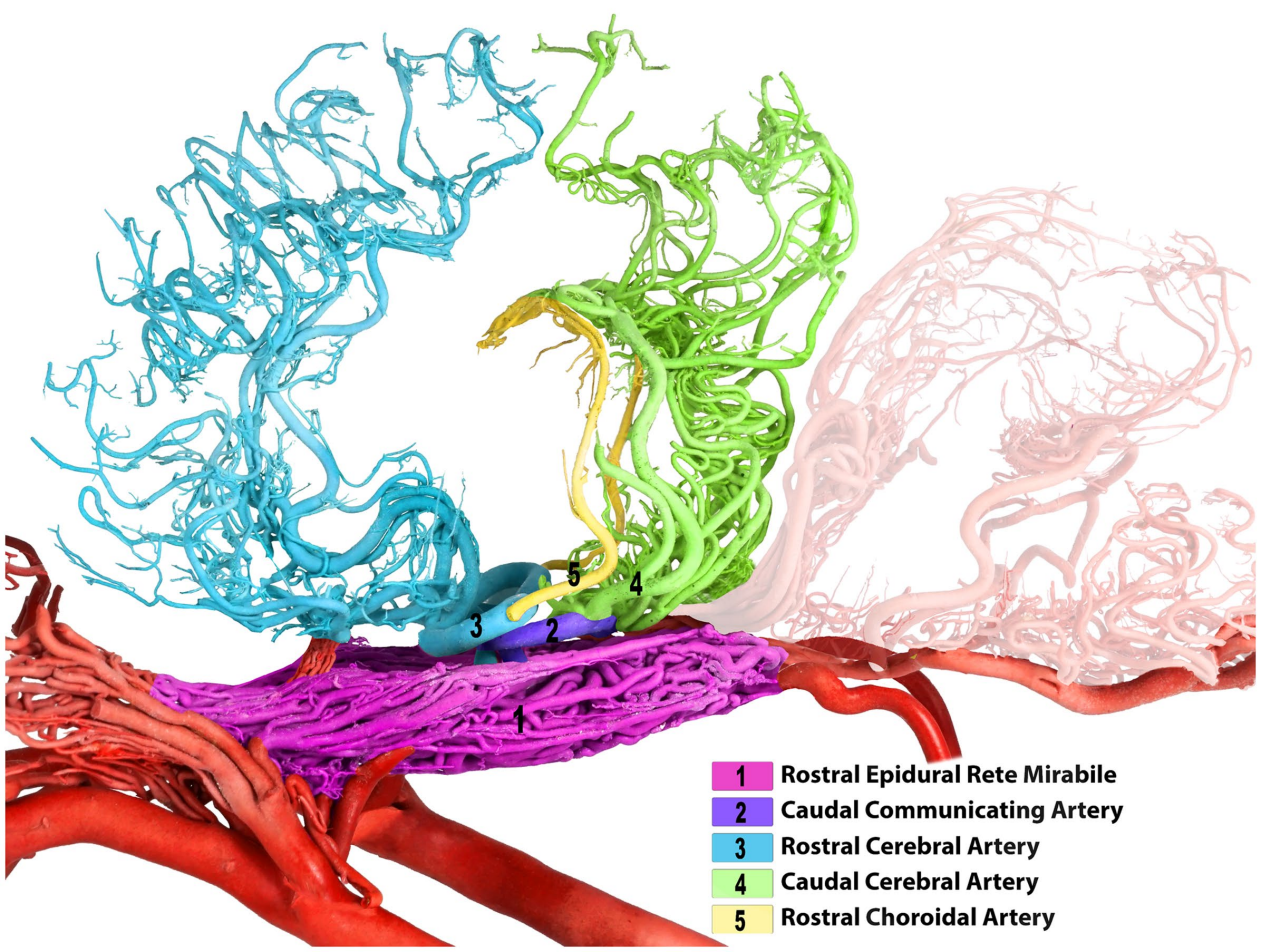
Left lateral view of the dromedary brain with vascular cast after removing both middle cerebral arteries for better clarity of the arterial course.

**Figure 3:**
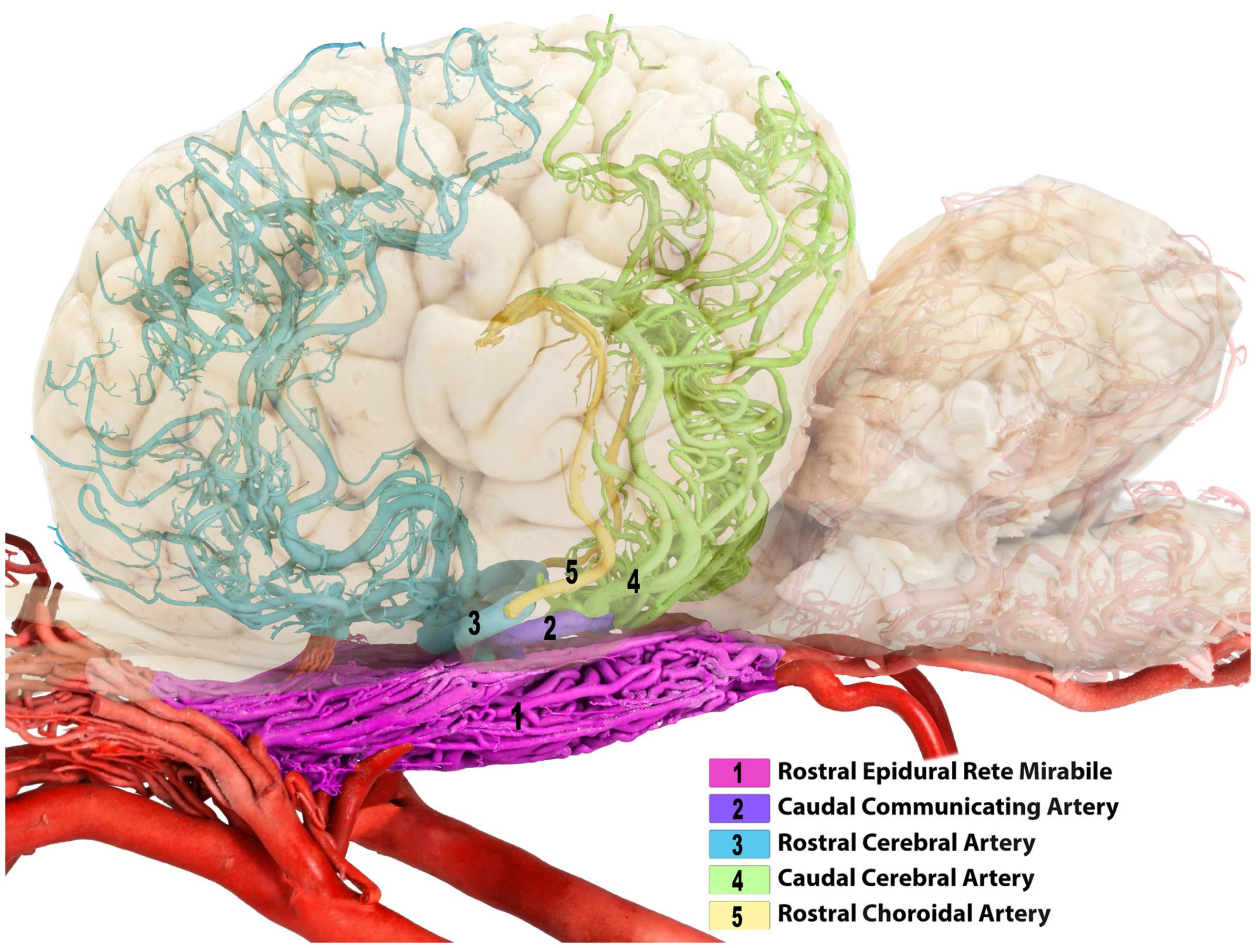
Composite image of the camel brain. The shadow of the brain is displayed, allowing visualization of its overall structure and highlighting the rostral and caudal cerebral arteries.

**Figure 4:**
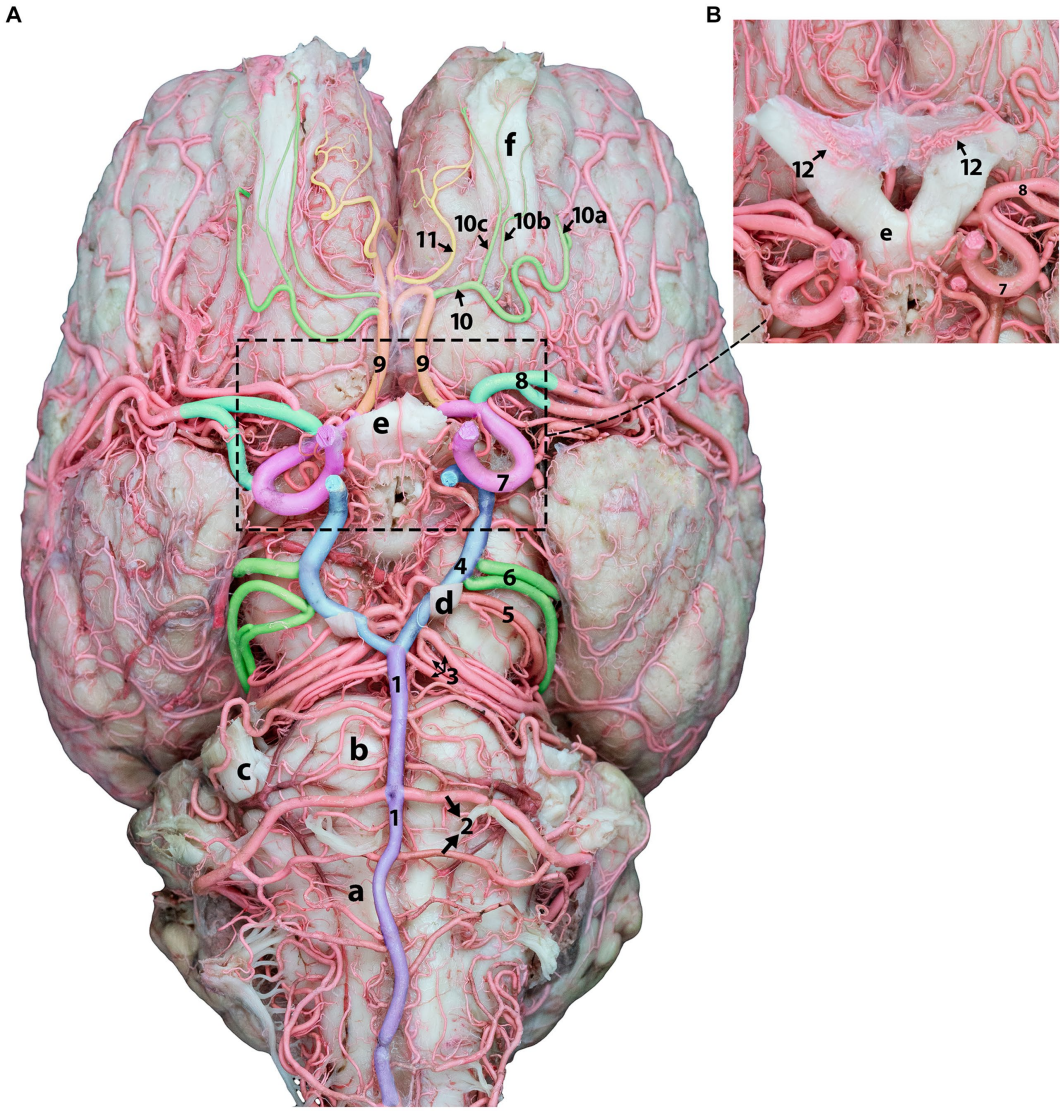
Ventral view of the dromedary brain and its blood supply. **(A)** Displays the ventral view of the camel brain after removing the optic nerves for better artery visualization. **(B)** Shows optic nerves and chiasmatic rete before removal.1, basilar artery; 2, caudal cerebellar artery; 3, rostral cerebellar artery; 4, caudal communicating artery; 5, caudal choroidal artery; 6, caudal cerebral artery; 7, rostral cerebral artery; 8, middle cerebral artery; 9, orbitofrontal artery; 10, rostral olfactory artery; 10a, lateral olfactory artery; 10b, middle olfactory artery, 10c, medial olfactory artery; 11, marginal artery; 12, chiasmatic rete; a, medulla oblongata; b, pons; c, trigeminal nerve; d, oculomotor nerve, e, optic chiasm; f, olfactory tract.

The rostral cerebral artery gives rise to its first branch, the rostral choroidal artery (Figures 1–3). The rostral choroidal artery follows a lateral course and then curves upward to reach and supply the choroid plexus of the third ventricle. During ascent, the rostral choroidal artery passed near the caudal cerebral artery (Figures 1–3). The second branch of the rostral cerebral artery was the middle cerebral artery. In most cases, the MCA originates from two locations on the rostral cerebral artery, which merge to form the main trunk of the MCA (Figures 1, 4).

**Figure 5:**
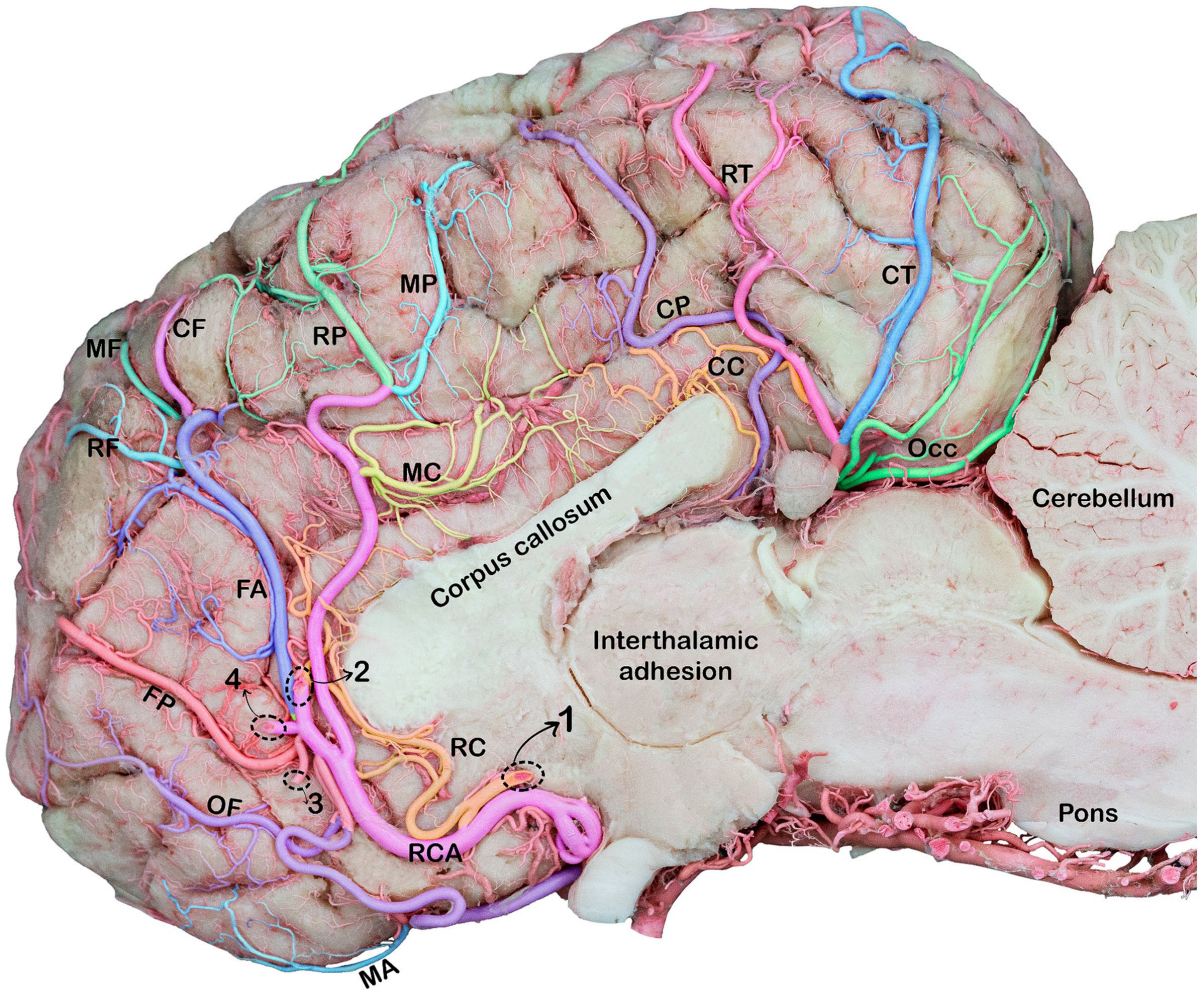
Medial view of the right cerebral hemisphere of the dromedary brain, indicating the branches of the rostral and caudal cerebral arteries. RCA, rostral cerebral artery; OF, orbitofrontal artery; MA, marginal artery; FP, frontopolar artery; FA, frontal artery; RF, rostral frontal artery; MF, middle frontal artery; CF, caudal frontal artery; RP, rostral parietal artery; MP, middle parietal artery, CP, caudal parietal artery; RC, rostral callosal artery; MC, middle callosal artery; CC, caudal callosal artery; RT, rostral temporal artery; CT, caudal temporal artery; OCC, occipital branches; Note: Dotted circles indicate a break in the branches of the rostral cerebral artery (RCA) that fuse to form a single trunk. 1, rostral callosal artery; 2, parietal artery; 3, frontopolar artery; 4, frontal artery.

The orbitofrontal artery originates from the rostral cerebral artery, which is dorsal to the optic chiasm. It then travels rostrally into the longitudinal fissure and curves around the subcallosal gyrus. Upon termination at the orbitofrontal cortex, it supplies the ectogenic gyri and medial surface of the frontal cortex (Figures 4, 5). Following its departure from the main stem of the orbitofrontal artery, the rostral olfactory artery (Figure 4) travels to the lateral rhinal sulcus. It travels toward the rostral portion of the lateral rhinal sulcus and gives several olfactory arteries that run along the olfactory tract and ascend toward the cribriform plate (Figure 4). They release smaller branches that wrap and curl around the olfactory tract. The marginal artery (Figures 4, 5) is a thin branch that originates from the orbitofrontal artery shortly after the rostral olfactory artery and extends in the rostral and dorsal directions. It supplies the medial surfaces of the frontal lobes, subcallosal gyrus, and genual and ectogenic gyri.

The rostral cerebral artery courses in the rostromedial direction until it reaches the beginning of the longitudinal fissure (Figures 4, 5). The rostral communicating artery is a short arterial bridge that connects the left and right rostral cerebral arteries (Figure 1). In our study, we observed that in six studied samples, at the level of the optic chiasma, the bilateral RCAs were interconnected via a short rostral communicating artery. In the remaining samples, no bridge connecting the two rostral cerebral arteries was observed because the two arteries converged medially prior to ascending along the longitudinal fissure.

**Figure 6:**
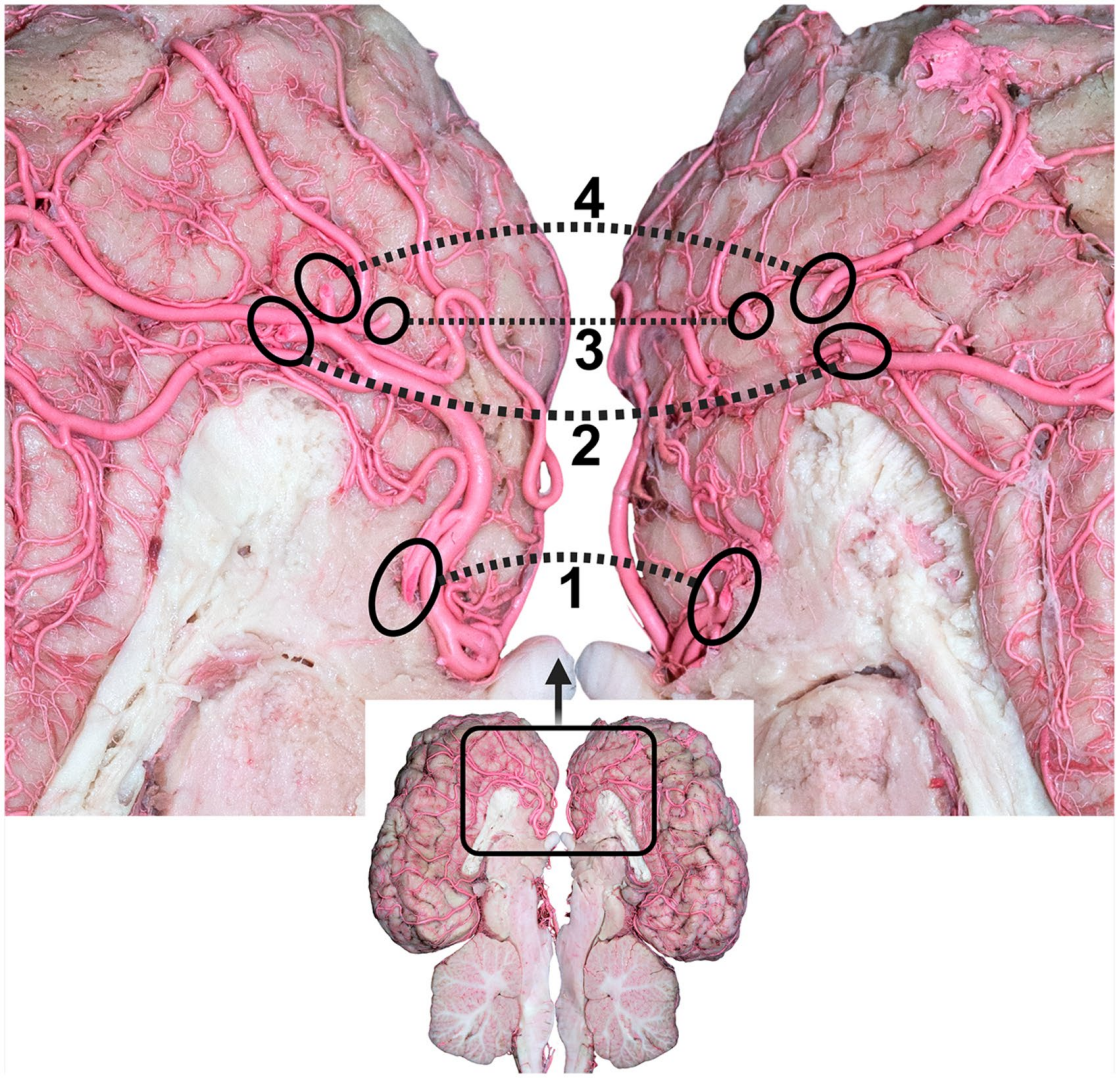
Dorsal view of the dromedary brain after cutting the brain through the longitudinal axis, indicating the branches of the rostral cerebral arteries. 1, rostral callosal artery; 2, parietal artery; 3, frontopolar artery; 4, frontal artery. Dotted circles indicate the origin of the branches of the rostral cerebral artery (RCA) to the other cerebral hemispheres.

Thereafter, the two rostral cerebral arteries follow a curved path around the genu of the corpus callosum. At this stage, the RCAs flow caudally on the medial surface of the cerebral hemispheres, giving rise to several cortical and central branches. The cortical branches supplying the medial surface of the parietal and frontal lobes of the cerebral hemisphere bear the names of their corresponding areas (parietal and frontal) (Figures 5, 6).

The frontopolar artery arises from the rostral cerebral artery, emerges close to the curve of the corpus callosum, and runs obliquely across the medial surface of the cerebral hemisphere toward the frontal pole (Figures 5, 6).

The frontal artery arises from the rostral cerebral artery and climbs through the genual sulcus to reach the frontal lobe (Figures 5, 6). The frontal artery extends from the genual sulcus to the rostral portion of the callosomarginal sulcus. It provides three distinct branches, called the rostral, middle, and caudal frontal branches, which supply the medial cortical surface of the frontal cortex and lobes (Figure 5). The rostral frontal branches supply blood to the rostral portion of the genual and post-cruciate gyri. The middle frontal branch supplies blood to the middle portion of the genual and post-cruciate gyri. The caudal frontal branch extends up to the ansate sulcus and supplies the caudal region of the post-cruciate gyrus and the rostral portion of the marginal gyrus (Figure 5).

The rostral cerebral artery then climbs the genual sulci to the callosomarginal sulcus and proceeds toward the parietal lobe, bifurcating into two distinct branches. These branches were identified as the rostral and middle parietal arteries that supply the medial surface of the parietal cortex (Figure 5). The rostral parietal branch travels in the direction of the ansate sulcus and extends from the rostral portion of the callosomarginal sulcus to that of the endomarginal sulcus. Its branches supply the cortical area beneath the endomarginal sulcus, rostral portion of the marginal gyrus, and splenial gyrus (Figure 5). The middle parietal branch extends from the callosomarginal sulcus and then ascends toward the initial segment of the middle to the splenial sulcus before continuing toward the middle endomarginal sulcus, supplying blood to the medial surface of the middle segment of the marginal and endomarginal gyri (Figure 5). Our observations revealed that the caudal parietal artery originates directly from the caudal cerebral artery. It traverses the caudal portion of the callosomarginal sulcus. It then ascends toward the middle segment of the splenial sulcus before continuing toward the endomarginal sulcus. The branches of this artery disperse across the surface of the final segment of the cingulate gyrus, splenial middle portion, and marginal gyrus (Figure 5).

Our findings revealed that the rostral cerebral artery gives rise to several short perforating arteries, called central branches, arising from the proximal surface of the rostral cerebral artery. They supply the deep cerebral structures, namely the hypothalamus, fornix, corpus callosum, posterior corpora quadrigemina, vermis, and associated structures.

The corpus callosum receives blood from the rostral, middle, and caudal callosal arteries. Presently, the rostral callosal artery originates from the rostral communicating artery. It then travels in a curved manner around the genu of the corpus callosum, providing a blood supply to the medial regions of the rostrum of the corpus callosum (Figure 5). The middle callosal artery arises from the rostral cerebral artery above the corpus callosum. It supplies the body of the corpus callosum, whereas the caudal callosal artery is derived from the caudal cerebral artery and supplies the splenium and caudal part of the corpus callosum.

Unlike the RERM, the chiasmatic rete connects the two rostral cerebral arteries to the external ophthalmic artery or rostral branches of the maxillary artery through a delicate mesh of anastomosing arteries close to the optic chiasma (Figure 4B).

The medial root of the internal ophthalmic artery, which consists of three or four thick branches emerging from the rostral cerebral artery, runs through the chiasmatic rete. The external ophthalmic artery is connected to the lateral root of a few smaller anastomosing arteries from the charismatic rete within the ophthalmic rete, which is located close to the rostral branches of the maxillary artery (Figure 4B).

Cortical and medullary arteries emerge from the caudal communicating artery. The cortical arteries supply blood to the parietal, temporal, and occipital lobes of the cerebral cortex. The brainstem and other structures deep within or ventral to the cerebrum receive supplies from the medullary branches. Typically, one or two thick arteries make up the arteries that supply the cortex. They originate from multiple points on the CCoA at the most curved caudal point of the CAC. These are the largest arteries that emerge from the caudal communicating artery near the base of the oculomotor nerve, commonly known as the caudal cerebral arteries (Figures 1–4).

**Figure 7:**
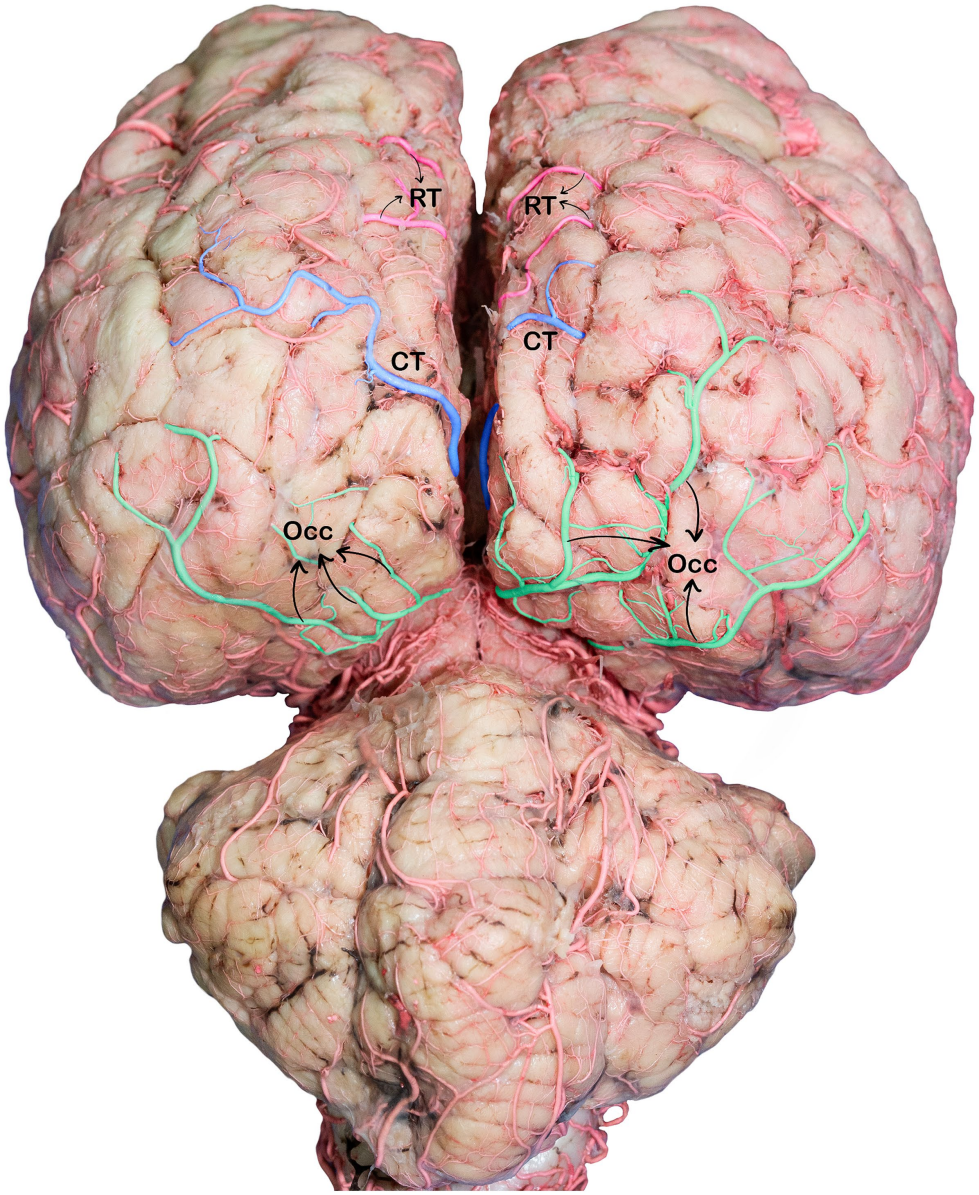
Caudodorsal view of the dromedary brain, indicating the branches of the caudal cerebral arteries. RT, rostral temporal artery; CT, caudal temporal artery; OCC, occipital branches.

The caudal cerebral arteries move laterally and ascend following the curves of the cerebral peduncle and corpora quadrigemina. As they ascend, they move medially toward the longitudinal fissure and approach their corresponding arteries dorsal to the quadrigeminal plate. Upon arrival at the longitudinal fissure, the artery proceeds dorsally and gives off branches that supply the medial surfaces of the temporal and occipital lobes, including the rostral and caudal temporal branches, as well as the lateral and medial occipital branches (Figure 5). The artery, which climbs the callosomarginal sulci and progresses toward the temporal lobe, is divided into two distinct branches. These branches were identified as the caudal and rostral temporal branches, which supply the medial cortical surface of the temporal cortex and descend at various levels (Figure 5). The rostral temporal branches climb the callosomarginal sulci and then ascend toward the splenial sulcus before continuing toward the endomarginal sulcus, supplying blood to the medial surface of the marginal and endomarginal gyri (Figure 5). The caudal temporal branches travel toward the endomarginal sulcus, giving off their first branch into the sulcus. The remaining branches then disperse across the surface of the endomarginal gyrus and the caudal segment of the marginal gyrus (Figures 5, 7).

The occipital branches of the caudal cerebral artery usually consist of the lateral and medial branches that descend at various levels. These branches supply the caudal segment of the endomarginal, marginal, and occipital gyri, as well as the medial surface of the occipital lobe (Figures 5, 7).

These cortical branches then turn outward, supplying to the lateral surfaces of the temporal and occipital lobes (Figures 5, 7). In addition to the temporal and occipital cortical branches of the caudal cerebral artery, we observed numerous tiny deep-penetrating branches (central branches) from the caudal cerebral artery supplying blood to the pineal gland, choroid plexus of the third ventricle, and the medial surfaces of the thalamus, hypothalamus, and associated structures.

## 4 Discussion

This study is the first to offer a comprehensive and detailed description of the origin, anatomical paths, and branching patterns of the rostral and caudal cerebral arteries in dromedaries. This study builds upon a recent extensive study of the origin, course, and branching patterns of the middle cerebral artery in dromedary camels (15).

To the best of available knowledge, no specific studies have addressed the role of the rostral and caudal cerebral arteries in supplying blood to animal brains. It is important to clarify that any comparisons made between the findings in camels and those in other animals pertain to the origin of the arteries, not their branches. No studies were found that specifically described or investigated the branching patterns of these arteries in other animals. Identifying and characterizing the branches of the rostral and caudal cerebral arteries in camels is challenging because of the lack of previous research. This study evaluates the courses and branches of the rostral and caudal cerebral arteries based on their respective supply (17).

The current study found that only four arteries, namely the right and left RCAs and the right and left CCoAs, arose dorsally from the RERM and penetrated the dura mater. These arteries form lateral walls and are major contributors to the cerebral arterial circle. The cerebral arterial circle is an important arterial structure located at the base of the brain and is responsible for regulating blood flow and maintaining blood pressure within the brain (9, 10, 18, 19). Some studies have indicated that the rostral cerebral artery and caudal communicating artery contribute significantly to the arterial circle of the brain and are branches of the terminal intracranial division of the internal carotid artery in camels (11, 20). Alsafy et al. (21) and Brudnicki (22) have reported that the internal carotid artery in goats bifurcates into the rostral cerebral and caudal communicating arteries. According to the study by Ozgel (23) in dogs and that by Ashwini et al. (24) in humans, cows, sheep, goats, and pigs, the internal carotid artery is divided into three main branches: rostral, middle, and caudal. However, Kapoor et al. (25) reported that in dogs, monkeys, goats, and sheep, the internal carotid arteries divide only into the rostral and middle cerebral arteries. In this study, no direct link was found between the internal carotid artery and arteries arising from the RERM. Instead, the internal carotid artery ended in the RERM after contributing to it. Kiełtyka-Kurc et al. (4) also reported similar findings in camels. In a previous study (12), we discovered that the RERM is formed by the internal carotid, middle meningeal, rostral branches of the maxillary, and external ophthalmic arteries. However, the RERM gives rise to only two arteries: the rostral cerebral and caudal communicating arteries. Therefore, it cannot be definitively stated whether these two arteries are the terminal branches of the internal carotid arteries.

Horses, dogs, cats, giraffes, and several other Bovidae, including ox, bantengs, yaks, and bison, have three segments of the RCA (6, 18, 26). The first segment ascends between the origin of the middle cerebral artery and the terminal division of the internal carotid artery. The second segment reaches the optic chiasma, and at the region of the ethmoidal fossa, the third segment of the rostral cerebral artery anastomoses with the internal ethmoidal artery, and this terminal segment passes through the longitudinal fissure of the brain, bends on the genu of the corpus callosum, and courses along the medial surface of the cerebral hemispheres (6, 18, 26).

This study revealed that the rostral cerebral artery gives rise to its initial branch, the rostral choroidal artery, followed by the middle cerebral artery. Kiełtyka-Kurc et al. (4) reported similar results in Bactrian camels. According to John et al. (27), in buffalo, the RCA was observed to split into two to three branches at the anterodorsal aspect of the medial surface of the frontal lobe. Our study revealed that the rostral cerebral artery sends several cortical branches to the medial surface of the frontal and parietal lobes. These branches are sufficiently long to supply the dorsal surfaces of the lobes. This pattern is consistent with existing research on the human brain, where the rostral cerebral artery generates several cortical branches, including the anterior, middle, and posterior frontal arteries, as well as the superior and inferior parietal arteries, corresponding to the area of supply (28, 29).

The orbitofrontal artery was observed to originate from the rostral cerebral artery dorsal to the optic chiasm. This finding is consistent with those reported by Gomes et al. (28) and Avci et al. (30) in humans. Furthermore, the rostral olfactory artery was observed to stem from the orbitofrontal artery. According to Gillilan (31), in pigs, the rostral cerebral artery follows a semicircular path toward the intercerebral fissure, whereas the major portion of the rostral division proceeds through the ethmoidal region and accompanies the olfactory nerve forward, branching off to the olfactory tract, olfactory bulb, piriform cortex, and tuberculum olfactorium.

The marginal artery was revealed to be a thin branch originating from the orbitofrontal artery shortly after the rostral olfactory artery, extending in the rostrodorsal direction. Some studies (22, 32, 33) have referred to the terminal parts of the rostral cerebral arteries as marginal arteries in sheep and goats.

The corpus callosum is an important commissural pathway connecting the two cerebral hemispheres. The corpus callosum was observed to receive blood from three main arteries: the rostral, middle, and caudal callosal arteries. Because the corpus callosum is supplied with blood through multiple arteries, infarction of the entire corpus callosum is rare (34). The rostral callosal arteries emerge from the rostral communicating arteries, the middle callosal arteries arise from the rostral cerebral arteries, and the caudal callosal arteries arise from the caudal cerebral arteries. In a study conducted by Takahashi et al. (34), the corpus callosum in humans was found to be supplied with blood by the anterior communicating, pericallosal, and posterior pericallosal arteries, which are branches of the anterior and posterior cerebral arteries. Several studies, including those by Jenke (35) in horses, Kanan (36) in dromedaries, Klein (37) in cats, and Brudnicki (22) in goats, have identified the terminal portion of the rostral cerebral artery as the artery of the corpus callosum.

The chiasmatic rete, a small network of fine arteries near the optic chiasma, was found to receive delicate internal ophthalmic arterial branches from the rostral cerebral artery. It has been found that the chiasmatic rete plays a key role in regulating the temperature of the orbital zone (38, 39). Camelids, sheep, and cows exhibit the chiasmatic rete (4, 6, 40, 41).

This rete is linked to the ophthalmic rete and situated in close proximity to the branches that emerge from the maxillary artery and contribute to the RERM. Other authors (4, 6, 42) identified the internal ophthalmic arteries in bovines and camels as thin arteries connected to the chiasmatic rete. In six of the examined samples, a connection was found between the two rostral cerebral arteries, known as the rostral communicating artery. This finding is consistent with that of a previous study on Bactrian camels by Kietyka-Kurc et al. (4). However, the rostral communicating artery displays significant variations in bovines and other camelids, such as guanaco and llama (4, 6). We believe that the presence of the rostral communicating artery may provide enhanced collateral circulation and protection against ischemic events in the event of arterial occlusion.

The CCA is among the most prominent branches of the cerebral arterial circle. It arises from the CCoA at the level of origin of the oculomotor nerve. Similar findings have been reported in dogs, foxes (43–45), horses, zebras, donkeys (43, 46), rabbits, hares (47, 48), camels, guanacos, llamas, cattle, bantengs, yaks, and bison (4, 6, 11), which branch off from the caudal communicating arteries.

Kiełtyka-Kurc et al. (20) reported that the CCA displayed vascular variations in fallow deer. The variation was either a branch of the RCA or emerged at the point where the internal carotid artery (ICA) divided. In the red-necked wallaby, the artery branches from the ICA (49). Similar findings have been reported in certain rodents (50, 51). Some rodents, including agouti and European beavers, have a CCA that branches from the terminal branches of the basilar artery (52, 53). In cattle, sheep, and pigs, the caudal division of the internal carotid artery turns caudally, followed by the formation of a large caudal cerebral artery (31). The basilar artery is divided into two caudal cerebral arteries in dogs and monkeys, each of which is joined to the corresponding internal carotid artery by a short slender posterior connecting artery (25).

However, this study indicated that the caudal cerebral artery originates from multiple points of the caudal communicating artery in the dromedary. Double caudal cerebral arteries have been observed in the llamas, Bactrian camels, and dromedaries (4). Godynicki and Wiland (54) identified comparable vascular types in roe deer, whereas Brudnicki (22) reported similar findings in domestic goats. Some Bovidae species, such as European bison, banteng, and domestic cattle, have two caudal cerebral arteries (6). According to Brown (50), bilateral caudal cerebral arteries result from the division of the middle cerebral artery, which participates in the formation of the arterial circle of the brain.

In some studies, the caudal cerebral artery has been identified as the caudal choroidal (55) and rostral cerebellar arteries (20). This study, however, observed a clear demarcation between the caudal and rostral cerebral arteries, with no apparent anastomosis. This finding contradicts earlier observations by Kiełtyka-Kurc et al. (4), who reported that CCAs anastomose with RCAs through the supplementary branches.

The rostral cerebellar and caudal cerebral arteries in camels were observed to have distinct proximal origins and routes, with each artery supplying separate brain regions. The epoxy cast models of the two arteries used in this study clearly distinguished their supply regions. They do not appear to anastomose with each other, which contradicts earlier findings suggesting that there may be communication pathways between the two arteries (4, 56).

The caudal cerebral arteries in the dromedaries were observed to move laterally and ascend close to the rostral choroidal artery, following the curvature of the cerebral peduncles and colliculi. They then move medially and approach their pair dorsal to the quadrigeminal plate of the midbrain, toward the longitudinal sulcus. Afterward, they travel medially and approach their partner, which is located adjacent to the longitudinal sulcus, dorsal to the quadrigeminal plate of the midbrain. The caudal cerebral artery in pigs circles the brainstem and distributes the posterior intercerebral fissure, which joins the fissure from the opposite side and forms a single caudal cerebral artery (31).

The course of caudal cerebral artery flow in pigs, sheep, and cattle has been documented by Gillilan (31). The caudal cerebral artery runs along the intercerebral fissure and supplies the caudal and medial surfaces of the hemispheres in pigs. Its cortical branch anastomoses dorsally with the ramus of the middle cerebral arteries and rostrally with the ramus of the rostral cerebral artery (31). In sheep, the caudal cerebral artery passes along the brainstem and extends to the caudal pole and medial surface of the cerebral hemisphere; a similar pattern has been observed in calves (31).

Upon arriving at the longitudinal sulcus, the caudal cerebral artery proceeds dorsally, makes a hairpin loop at the sulcus, and produces several cortical branches that supply the medial surface of the temporal and occipital lobes of the cerebral hemisphere. These branches then turn outward and supply to the lateral surfaces of the temporal and occipital lobes. In buffalo, the CCA was divided into a caudal branch and rostral branches. The caudal branch supplied the posterior and inferior aspects of the occipital lobe. While the rostral branches supplied the caudomedial area of the occipital lobe (27). The same observation has been made in humans, showing that several cortical branches originate from the posterior cerebral artery, including the anterior temporal, posterior temporal, medial occipital, and lateral occipital arteries (57). It should be emphasized that the comparisons presented in this study between the branches of the rostral and caudal cerebral arteries in camels and humans, as no prior research on these arteries in animal brains has been conducted.

## 5 Conclusion

This study provides a comprehensive analysis of the origin, pathways, and patterns of blood supply by the rostral and caudal cerebral arteries and their branches in camels. Our findings contribute significantly to the existing scientific literature by filling a critical knowledge gap in this area. Using advanced casting techniques to analyze the cerebral arteries, this study generated accurate three-dimensional representations of the minor branches that may otherwise have been overlooked during the dissection of the cerebral arteries, enhancing our understanding of these vessels. While this study provides valuable insights into the vascular architecture of the rostral and caudal cerebral arteries in camels, it does not address the functional aspects of cerebral blood flow, which would require further investigation using complementary techniques.

## Data availability statement

The raw data supporting the conclusions of this article will be made available by the authors, without undue reservation.

## Ethics statement

The animal study was approved by Animal Research Ethics Committee, United Arab Emirates University. The study was conducted in accordance with the local legislation and institutional requirements.

## Author contributions

AA: Conceptualization, Data curation, Formal analysis, Funding acquisition, Investigation, Methodology, Project administration, Resources, Software, Supervision, Validation, Visualization, Writing – original draft, Writing – review & editing. RB: Data curation, Formal analysis, Investigation, Writing – original draft, Writing – review & editing.
